# Measuring the Conformational Distance of GPCR-related Proteins Using a Joint-based Descriptor

**DOI:** 10.1038/s41598-017-15513-3

**Published:** 2017-11-09

**Authors:** Jayaraman Thangappan, Bharat Madan, Sangwook Wu, Sun-Gu Lee

**Affiliations:** 1 Department of Chemical Engineering, Pusan National University, Busan, 609-735 Republic of Korea; 2 Department of Physics, Pukyong National University, Busan, 608-737 Republic of Korea

## Abstract

Joint-based descriptor is a new level of macroscopic descriptor for protein structure using joints of secondary structures as a basic element. Here, we propose how the joint-based descriptor can be applied to examine the conformational distances or differences of transmembrane (TM) proteins. Specifically, we performed three independent studies that measured the global and conformational distances between GPCR A family and its related structures. First, the conformational distances of GPCR A family and other 7TM proteins were evaluated. This provided the information on the distant and close families or superfamilies to GPCR A family and permitted the identification of conserved local conformations. Second, computational models of GPCR A family proteins were validated, which enabled us to estimate how much they reproduce the native conformation of GPCR A proteins at global and local conformational level. Finally, the conformational distances between active and inactive states of GPCR proteins were estimated, which identified the difference of local conformation. The proposed macroscopic joint-based approach is expected to allow us to investigate structural features, evolutionary relationships, computational models and conformational changes of TM proteins in a more simplistic manner.

## Introduction

Transmembrane (TM) proteins are essential in cellular and biochemical processes that are related directly to the external environment. TM proteins serve as the primary targets of medicinal drugs because of their important functional activities, such as signal transduction^1^, ion channeling^2,3^, energy metabolism^4^, and drug recognition^5^. TM proteins can be divided into two types: α-helical and β-barrels. In particular, α-helical proteins are the major category of TM proteins that are present in the inner membrane of bacterial cells and the plasma membrane of eukaryotic cells. Approximately 27% of proteins are estimated to be α-helical TM proteins in humans^6^. The structures of helical TM proteins are strongly related to their physical properties, such as folding, stability, and functions^7–9^. Their structures also provide information about how TM proteins have evolved and connected with each other^10^. Therefore, a study on the structural or conformational features of helical TM proteins has been an important issue. For example, various studies on their TM topology^11–14^, helix-helix packing pattern^15–20^, and structural diversity^21–25^ have been performed.

Measuring the structural distance or difference in proteins is crucial^26^. This is strongly related to the classification of proteins in nature, prediction of the protein structures, and the design of artificial proteins. Various approaches have been developed to measure the structural distance of proteins^27–29^. They are generally based on a microscopic description of the protein structures. A representative example is to estimate the structural distance of proteins using the Cα atom-based RMSD (Root Mean Square Deviation)^30–33^. Indeed, such microscopic descriptor-based approaches are effective in measuring the structural distance of proteins at the atomic level. On the other hand, the structural distance or difference in proteins can be measured on the macroscopic level^34,35^. Quantifying the distance of protein structures based on the topology of secondary structures is a representative example of the macroscopic approach^36,37^. When dealing with large-scale proteins, such as multi-protein complexes or TM proteins, it is advantageous to use macroscopic approaches, even though they cannot provide detailed information on the atomistic scale. Despite the loss of information on the atomistic level, they allow an examination of the conformational features of proteins in a more simplistic and effective manner.

Recently, we proposed a new macroscopic descriptor of protein structures, called joint-based descriptor^38^. The descriptor uses the joints of secondary structures, such as α helices, β sheets, and loops as the basic constituents. In that descriptor, the dihedral angles of the joints are effective in defining the conformation of proteins. In that study, the approach was applied successfully to investigate the conformational features of the TM proteins by analyzing a dataset of non-homologous TM proteins. For example, the allowed and disallowed regions of their joint-based dihedral angles were examined, which provided information on the possible conformational space of the helical arrangement in TM proteins. Further analyses not only identified the common patterns of helical arrangement and extension, but also detected some geometrically symmetric protein pairs in a non-homologous TM dataset.

In this study, a joint-based descriptor was applied to measure the conformational distance of helical TM proteins on a macroscopic level. The basic strategy was to identify the joint-based dihedral angles specific to a TM protein family, and estimate how far the joint-based dihedral angles of an interesting target TM protein deviate from the identified angles of the TM protein family. Here, the strategy was implemented to measure the conformational distance between the GPCR A protein family and its related structures. The GPCR A protein family, which is one of the largest 7TM families, engages in most of the signaling activities and is a major target for drug discovery^39,40^. More specifically, the following three independent case studies were performed: (i) the approach was applied to identify how far the global and local conformations of the 7TM proteins in the PDB database are from the GPCR A family; (ii) the approach was used to validate the computational models of the GPCR structures at the joint-based coordinate level, and (iii) the approach was applied to study the conformational difference between the active and inactive states of the GPCR proteins.

## Results

### Macroscopic description of the 7TM structure using a joint-based descriptor

**Figure 1:**
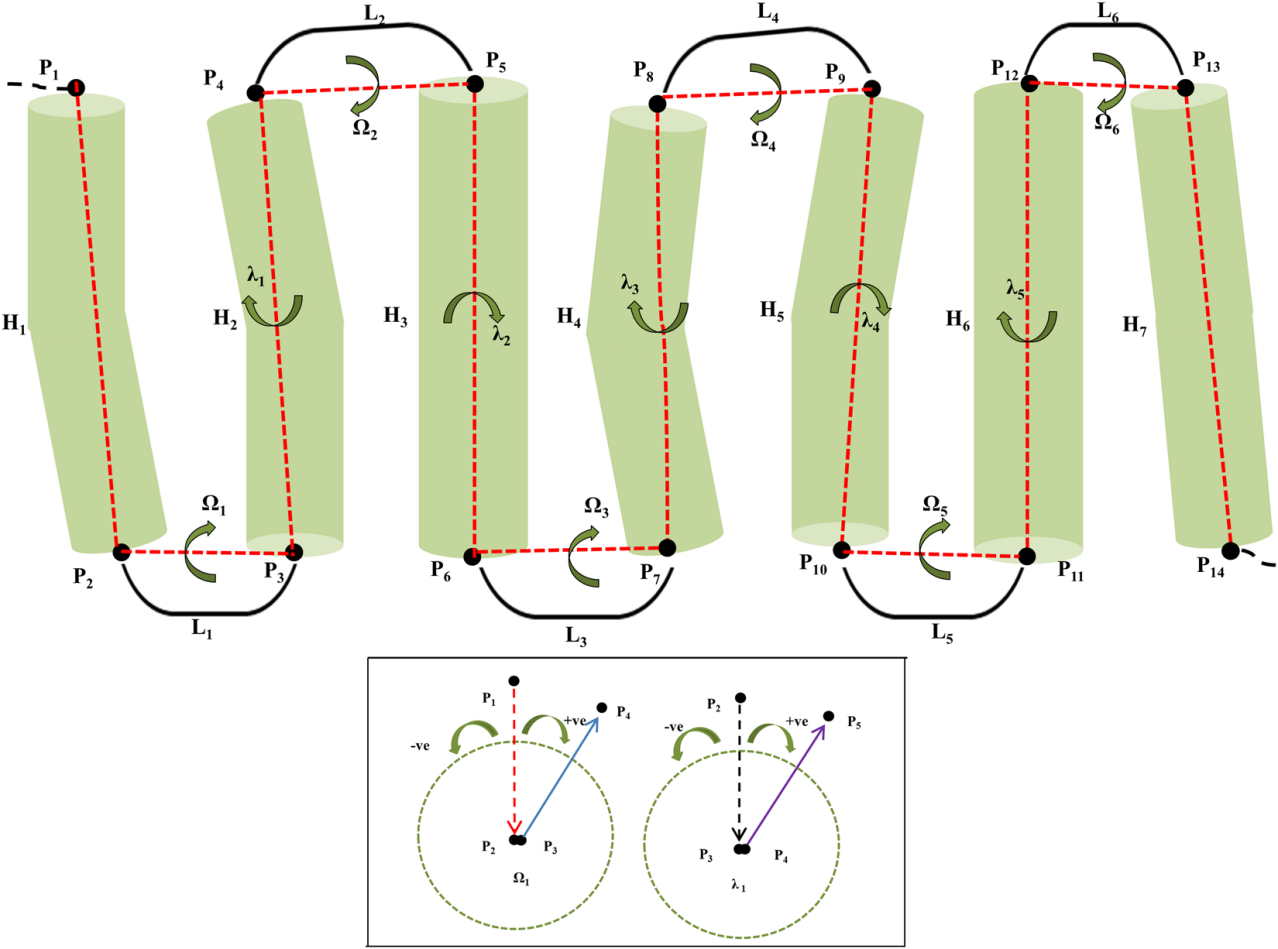
Joint-based description of 7TM protein structure. H_1_ to H_7_ are helices, L_1_ to L_6_ are loops, and P_1_ to P_14_ are joint points. Ω-type dihedral angles, such as Ω_1_, are defined by the four joint points in the Helix-Loop-Helix, such as P_1_, P_2_, P_3,_ and P_4_. The λ-type dihedral angles, such as λ_1_, are defined by the four joint points in the Loop-Helix-Loop, such as P_2_, P_3_, P_4,_ and P_5_. The figure presents the projection for ideal 7TM through Ω_1–6_ and λ_1–5_. The inset shows the example of the assignment of the positive and negative signs for dihedral angles using the projections for Ω_1_ and λ_1_, where the positive (+) and negative (−) signs represent the clockwise and counter-clockwise angles, respectively.

The joint-based descriptor defines a protein conformation through the dihedral angles of the joints of secondary structures, and the details of the descriptor were introduced in the previous report^38^. This section briefly reviews how the descriptor is applied to define TM conformations using the typical structure of a 7TM protein, which displays 7TM helices and 6 loops (Fig. 1). To present the structure based on the joint approach, a set of joints associating the helices and loops are selected. In particular, the C-alpha carbons of the starting and ending residues of each TM helix are considered as structural joining points and employed as the structural elements of a protein structure. Fourteen joint points (P_**1–14**_) can be assigned to a 7TM protein composed of seven helices (H_**1–7**_) and 6 loops (L_**1–6**_). The first dihedral angle involving four joints (P_**1–4**_) can be determined by measuring the angle between two planes made by (P_**1–3**_) and (P_**2–4**_). Similarly, the second dihedral angle can be measured by relating the structural joints (P_**2–5**_), and the (P_**3–6**_) joints are used to determine the third, and so on. The dihedral angles are classified into two types: Ω and λ types. The dihedral angle determined by the four joints in the Helix-Loop-Helix, such as the first and third dihedral angles, corresponds to the Ω type. In a similar manner, the dihedral angle determined by the four joints in a Loop-Helix-Loop, such as the second and fourth dihedral angle, is designated as the λ type. For the dihedral angles, the clockwise angle (from 0° to 180°) is assigned a positive value and the counter-clockwise angle is assigned a negative value (from 0° to −180°), as shown in the inset in Fig. 1. The conformation of a 7TM protein can be represented by a set of two types of dihedral angles, i.e., Ω_1_ to Ω_6_ and λ_1_ to λ_5_, at the macroscopic level. The detailed account to define the structural joints and the dihedral angles of the 7TM proteins used in this study are described in ***Methods***.

### Strategy to measure global and local distances of a 7TM protein from GPCR A family

As mentioned in the ***Introduction***, the primary objective of this study was to demonstrate how the joint-based descriptor can be applied to measure the conformational distance of an interesting 7TM protein from the GPCR A family. For this, a scoring function called the J-score was devised to quantify the differences between the dihedral angles of the joints specific to the GPCR A family and the corresponding dihedral angles of an interesting 7TM protein. This section describes how the joint-based dihedral pattern for the GPCR A family was obtained and how the J-score was defined. A strategy to measure the global and local distances between GPCR A family and a target 7TM protein is also proposed based on the estimated J-scores.

The first step to obtain the dihedral angle pattern specific to the GPCR A family was to select the representative proteins from the GPCR class A family proteins in the Protein Data Bank (PDB). For this, at least one receptor member type protein with high resolution was selected from each subfamily of the GPCR A family, which formed a non-redundant dataset composed of 27 proteins. The detailed procedure to obtain the 27 proteins is described in the ***Methods*** section. The proteins in the dataset were analyzed using the joint-based descriptor, which provided the 11 dihedral angles, as shown in Fig. 1 for each protein. SI Table 1 lists the PDB ID codes, subfamily types, and 11 dihedral angles of the 27 proteins. The mean and standard deviation (SD) of each dihedral angle estimated from SI Table 1 and are summarized in Table 1. A set of the estimated mean values for the 11 type dihedral angles, i.e., Ω_1_ to Ω_6_ and λ_1_ to λ_5_, was defined as a specific dihedral angle pattern for the GPCR A family.

**Table 1:** Mean and Standard Deviation of the dihedral angles for 27 GPCR_A structures.

Dihedrals	Specified Range	Mean	SD
Ω_1_	≥−22 to ≤−5	−14	±4
Ω_2_	≥−22 to ≤−8	−16	±4
Ω_3_	≥10 to ≤29	17	±4
Ω_4_	≥–29 to ≤20	−10	±13
Ω_5_	≥−29 to ≤−6	−18	±5
Ω_6_	≥−23 to ≤3	−5	±7
λ_1_	≥−134 to ≤−62	−101	±20
λ_2_	≥73 to ≤163	128	±19
λ_3_	≥−31 to ≤51	12	±22
λ_4_	≥−83 to ≤−4	−37	±17
λ_5_	≥−173 to ≤−109	−147	±20

Two types of J-score were devised to measure the local and global conformational distances between a target 7TM protein and GPCR A family. To measure the local conformational distance, the typical Z-score^41–43^, which suggests how far the observed value is away from the mean value by the number of SD, was employed and called J_i_, i.e., the J-score for the dihedral angle, i. Equation (1) defines J_i_, where X_i_ is Ω_i_ or λ_i_ for a target protein. μ_i_ and σ_i_ are mean and SD of each Ω_i_ or λ_i_ for GPCR A family in Table 1, respectively. The J_i_ presents how much the dihedral angle i of the target TM protein deviates from the mean dihedral angle i of the GPCR A family. To measure the global conformational distance, the J-scores for the 11 dihedral angles were normalized by the root mean square, called J_tot_ (Equation (2), N = 11 for 7TM protein). J_tot_ denotes how much the overall dihedral angle pattern of a target 7TM protein deviates from the overall dihedral angle pattern specific to the GPCR A family determined by the set of 11 mean dihedral angles.

1 $${{\bf{J}}}_{{\bf{i}}}=\frac{|{\bf{X}}{\bf{i}}-{\boldsymbol{\mu }}i|}{{\boldsymbol{\sigma }}{\bf{i}}}\,(|{\bf{X}}{\bf{i}}-{\boldsymbol{\mu }}i|\le {180}^{\circ }),\,{\bf{o}}{\bf{r}}\,\frac{\{{360}^{\circ }-|{\bf{X}}{\bf{i}}-{\boldsymbol{\mu }}i|\}}{{\boldsymbol{\sigma }}{\bf{i}}}\,(|{\bf{X}}{\bf{i}}-{\boldsymbol{\mu }}i| > {180}^{\circ })$$

2 $${{\bf{J}}}_{{\bf{tot}}}=[\sqrt{\frac{\sum {({{\bf{J}}}_{{\bf{i}}})}^{2}}{{\bf{N}}}}]$$

The calculated J-scores are interpreted in two ways. The first is a qualitative interpretation that a target protein is structurally closer to the GPCR A family as the measured J-score becomes smaller and more distant with increasing score. The other is a quantitative interpretation based on the values of the J-scores. For this, a set of J-scores for the selected 27 GPCR A family proteins are used as a reference. When the J-score of a target protein is in the range of J-scores for the reference set, the conformation of the target protein is considered to be “GPCR A *family*-*like*”. When the J-score of a target protein is more than the maximum value for the reference set, the target protein is classified as a “GPCR A *family*-*near*” or “GPCR A *family*-*far*” protein depending on its J-score. In this grouping, the score of 4 is used as a criterion, which is generally used to distinguish outliers in the Z-score statistics^44^. In summary, the target proteins are classified into the “GPCR A *family*-*like*”, “GPCR A *family*-*near*”, and “GPCR A *family*-*far*” when 0 ≤ J_score_ ≤ J_max_ of the reference set, J_max_ of reference set <J_score_ ≤ 4, and J_score_ > 4, respectively.

#### Measurement of conformational distance between GPCR A and other 7TM proteins

A structural comparison between protein families or superfamilies provides information on how the proteins have been evolved structurally and functionally^45–48^. In addition, it can be applied to many areas of structural bioinformatics, including homology modeling, fold recognition, and structural genomics^49^. GPCR A family belongs to the rhodopsin-like superfamily in 7TM fold. As a case study, the global and local structural distances between the GPCR A family and other proteins sharing common 7TM topology were determined by measuring and comparing their J-scores. As mentioned in the previous section, all types of J-scores for the 27 GPCR A family proteins were measured (SI Table 2) and used as a reference to analyze the data.

**Figure 2:**
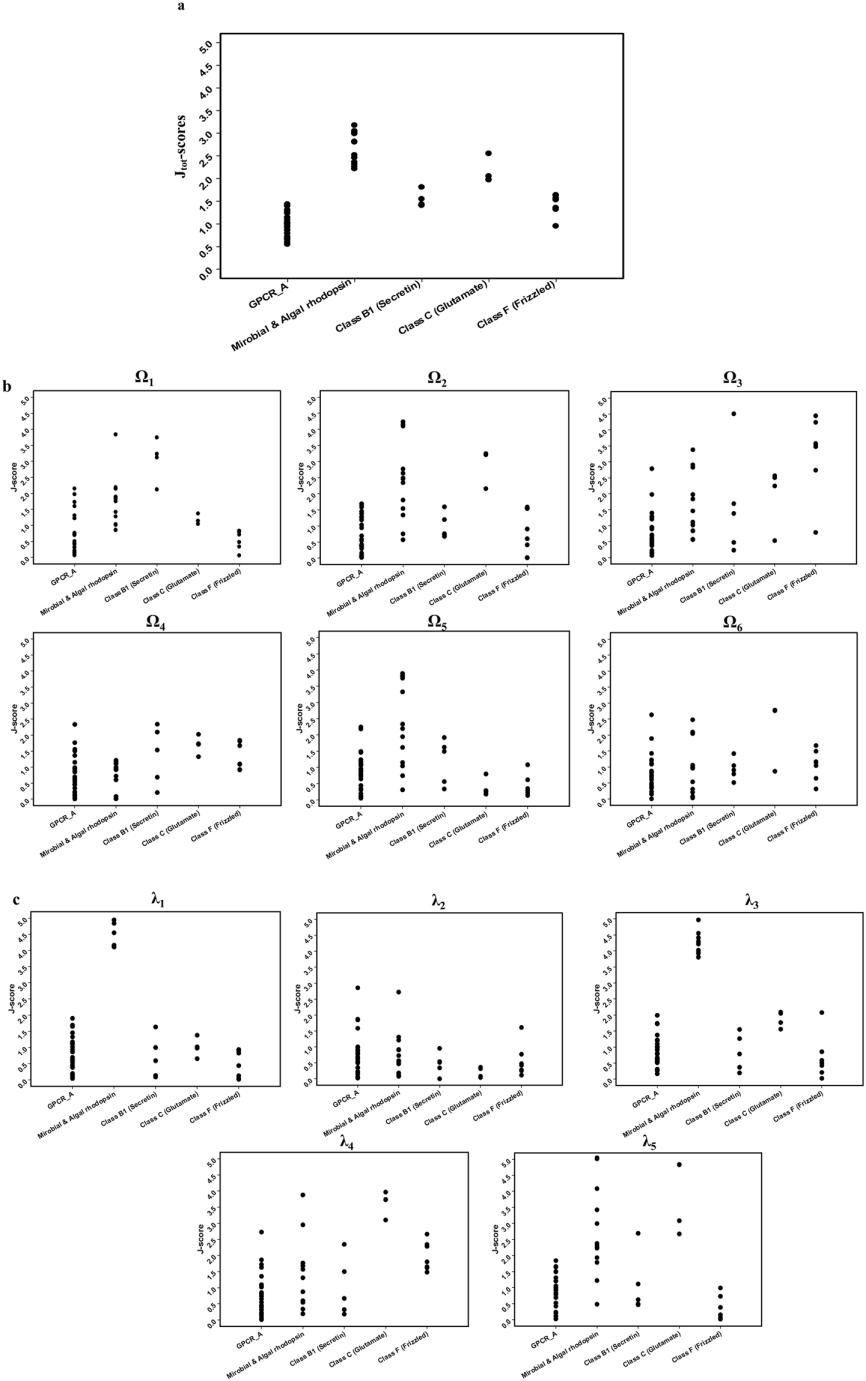
J-scores of the rhodopsin-like superfamily proteins in comparison with GPCR A family. Family names are presented in x-axis and their J-scores are presented in y-axis. (**a**) J_tot_-scores (**b**) J_i_-scores for individual omega (Ω_1–6_) angles, and (**c**) J_i_-scores for individual lambda (λ_1–5_) angles.

First, the conformational distances of the proteins in the rhodopsin-like superfamily from GPCR A family were evaluated. The rhodopsin-like superfamily contains 4 different families other than the GPCR A family, i.e., Microbial and Algal rhodopsin, Class B (Secretin), Class C (Glutamate), and Class F (Frizzled). All the non-redundant proteins of the 4 families in the PDB were selected, and their joint-based dihedral angles and J-scores were quantified, as shown in SI Tables 3 and 4, respectively. Figure 2(a) shows the measured J_tot_-scores of the 4 families with the reference score of the GPCR A family. The J_tot_-scores of the proteins belonging to the Microbial and Algal rhodopsin family and GPCR Class C family (Glutamate) were clearly higher than those of the GPCR A family proteins, whereas the J_tot_-scores of GPCR class B (Secretin) and GPCR class F (Frizzled) family proteins were very close to the J_tot_-scores of the GPCR A family proteins. These results suggest that the proteins in the Microbial and Algal rhodopsin family and GPCR Class C (Glutamate) family are relatively distant from the GPCR A family in the global conformation compared to the GPCR class B (Secretin) and GPCR class F (Frizzled) family proteins. On the other hand, the J_tot_-scores of the four families were all less than 4, which suggests that there are no proteins classified into “GPCR A *family*-*far*” in terms of global conformation. To examine their local conformational distances, the J_i_-scores for the individual Ω angles or λ angles were also compared (Fig. 2(b) and (c)). The data shows that most of the J_i_-scores for GPCR class B (Secretin) and GPCR class F (Frizzled) family proteins are closer to those of the GPCR A family proteins compared to the other two protein families. This suggests that the two family proteins have a similar conformation to the GPCR A family proteins in the local conformation. Most of the J_i_-scores of the four family proteins were less than 4, indicating that local conformations of the proteins are in the regions of “GPCR A *family like*” or “GPCR A *family*-*near*”. These results are somewhat consistent with the analytical results of the global conformation study, but some distinct features could be detected in this local conformation study as follows. Obviously, the J_i_-scores of λ_1_ and λ_3_ for the Microbial and Algal rhodopsin family proteins were higher than those of the GPCR A family proteins. In addition, they were mostly in the region of “GPCR A *family*-*far*”. For the GPCR Class C (Glutamate) family proteins, their J_i_-scores for Ω_2_, λ_4_, and λ_5_ were higher than the respective J_i_-scores of the GPCR A family proteins, and they were in the region of “GPCR A *family*-*near*”. These results denote the local dihedral angles that contribute to the global conformational distances between the two families and the GPCR A family. On the other hand, the J_i_-scores of the four family proteins for the Ω_4_, Ω_6_, and λ_2_ were all in the range of scores for the GPCR A family, i.e. “GPCR A *family*-*like*” region. This suggests that the proteins in the rhodopsin-like superfamily maintain a well-conserved conformation in those dihedral angles.

**Figure 3:**
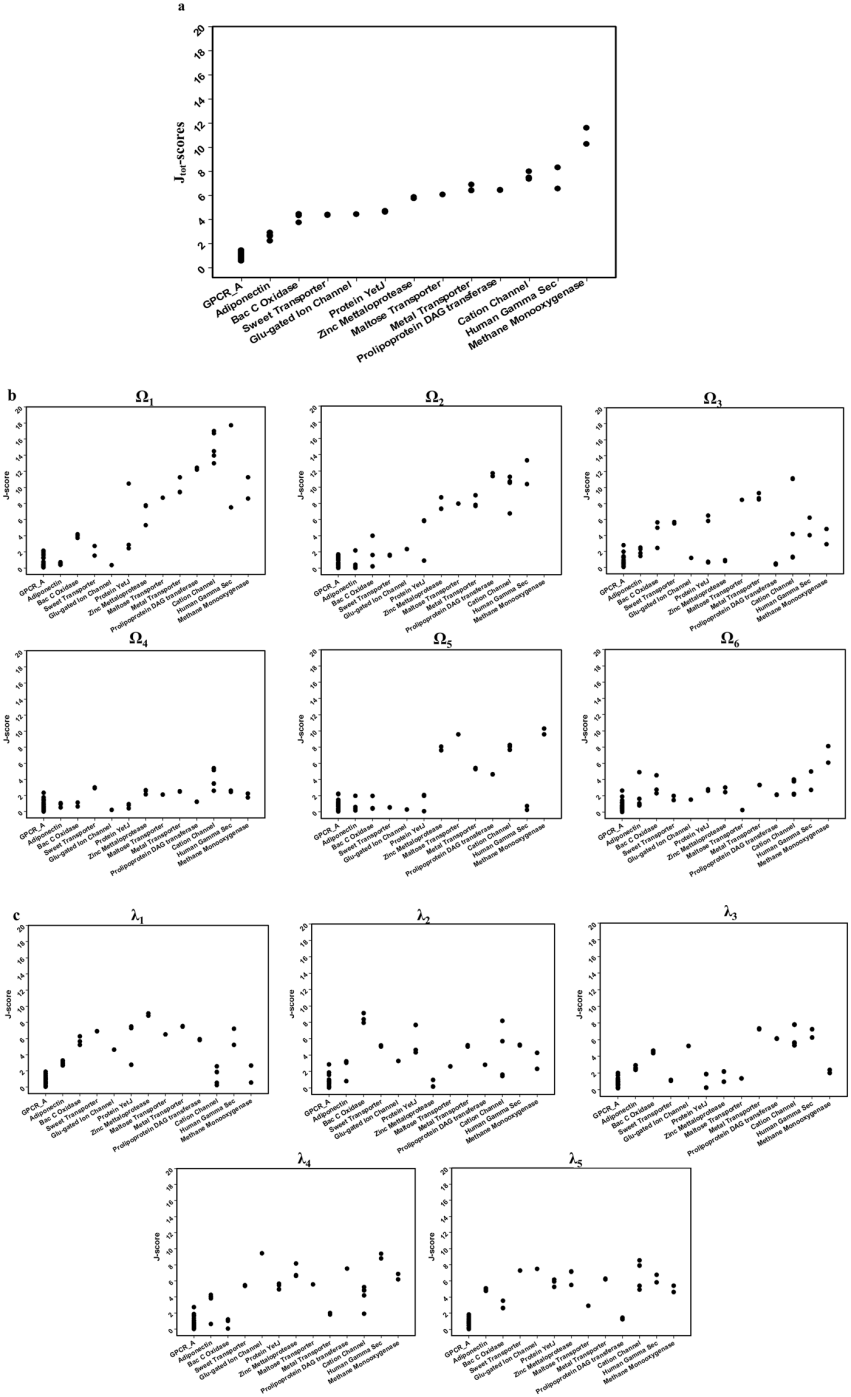
J-scores of the superfamilies in 7TM fold in comparison with GPCR A family. Superfamily names are presented in x-axis and their J-scores are presented in y-axis. (**a**) J_tot_-scores (**b**) J_i_-scores for individual omega (Ω_1–6_) angles, and (**c**) J_i_-scores for individual lambda (λ_1–5_) angles.

The conformational distances of the proteins in different 7TM superfamilies from the GPCR A family were quantified. In 7TM fold, there are 13 different superfamilies. The available non-redundant proteins in the superfamilies were selected from PDB, analyzed by the joint-based descriptor, and their J-scores were estimated (SI Tables 5 and 6). Figure 3(a) shows the J_tot_-scores for the GPCR A family proteins and proteins in the different superfamilies in the 7TM fold. The J-scores of all the superfamilies were higher than the scores for the GPCR A family proteins. No proteins were observed in the region of “GPCR A *family*-*like*”. Only the adiponectin superfamily proteins showed the J_tot_–score of “GPCR-A *family*-*near*” region. The J_tot_–scores for other superfamily proteins were observed in the region of “GPCR-A *family*-*far*”. In particular, the methane monooxygenase superfamily proteins showed the highest J_tot_-score. These results suggest that the proteins in the different superfamilies do not share the conformation with the GPCR A family globally at the joint-based coordinate level. The J_i_-scores for individual dihedral angles were also measured and compared (Fig. 3(b) and (c)). In this local conformational level, some superfamilies share a local conformation with the GPCR A family proteins. For example, the Adiponectin, Bacterial Cytochrome C oxidase, Sweet transporters, Glutamate Ion Channel, Protein Yet J superfamilies showed J_i_-scores for Ω_5_ in the range of the GPCR A family proteins. Interestingly, the J_i_-scores for Ω_4_ were lower than 4 and in the regions of “GPCR A *family*-*like*” or “GPCR A *family*-*near*” for most proteins except a few proteins in the Cation Channel superfamily. This suggests that the Ω_4_ dihedral angle is relatively well-conserved compared to other dihedral angles in the 7TM proteins. The Ω_6_ angle is the second well-conserved dihedral angle in 7TM proteins with a low J_i_-score among entire superfamilies.

Overall, the conformational distance of the GPCR A family and other 7TM proteins were measured based on the joint-based descriptor. The analysis allowed the distant and close families or superfamilies to the GPCR A family to be distinguished at a global conformation level. In addition, the conserved and diverse dihedral angles of the joint points in rhodopsin-like superfamily and in 7TM fold proteins could be identified. The above analyses showed the analytical results based on the Ω and λ angles. As reported previously^38^, the dihedral angles are related directly to the arrangement and extension of helices in the membrane. These results are interpreted in terms of the helical arrangement and extension pattern in the ***Discussion*** section.

### Conformational validation of computational models for human GPCRs

Many TM protein structures still remain unexplored because of the difficulty in their crystallization. Therefore, computational structural modeling is believed to be an alternative tool to identify the unknown structures^50–53^. In particular, a number of approaches to model the GPCR structures from sequences were developed due to the biological importance and profound effect of GPCR proteins in drug discovery and translational medicine. One of the most efficient modeling methods for GPCR is the GPCR I-TASSER method^54^, which is a hybrid method combining threading, *ab initio* folding and experimental data for the 3D structure of GPCR proteins. The protocol was used to construct a GPCR HGmod database, including the 3D structural models of almost 1000 of human GPCR candidates^54^. In this study, a set of the computational models in the database was analyzed by the joint-based descriptor, and their J-scores were measured to validate the quality of the models based on the conformational features of the known 27 GPCR protein A family proteins.

**Figure 4:**
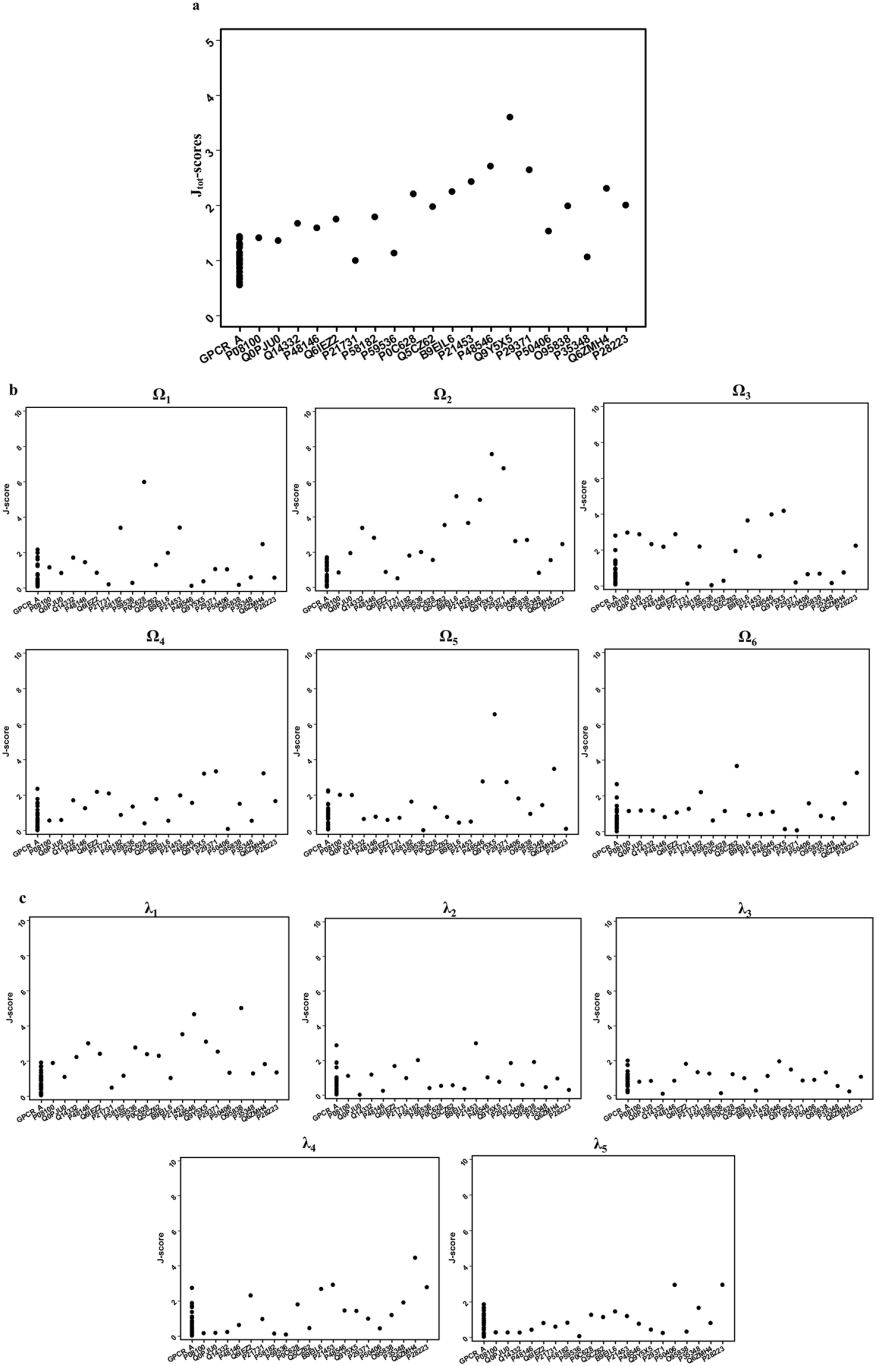
J-scores of the computational models of GPCR proteins obtained from the HGmod database in comparison with GPCR A family. 20 HGmod GPCR Model IDs are in x-axis, and their J-scores are in y-axis. (**a**) J_tot_-scores (**b**) J_i_-scores for individual omega (Ω_1–6_) angles, and (**c**) J_i_-scores for individual lambda (λ_1–5_) angles.

From the GPCR HGmod database^54^, 20 computational models were selected randomly, and their J-scores considering a total of 11 dihedral angles and individual angles were calculated. SI Tables 7 and 8 list the analyzed dihedral angles and J-scores of the 20 models, respectively. The J-scores were compared with those of the 27 GPCR A family proteins (Fig. 4). Among the 20 computational models, 6 models (*Opsin receptor*, *Opsin 1 receptor*, *Thromboxane receptor*, *Taste receptor type 2*, *5*-*hydroxytryptamine receptor 6 and Alpha*-*1A adrenergic receptor*) showed J_tot_-scores in the region of the “GPCR A *family*-*like*” conformation, and the other 14 models showed J_tot_-scores corresponding to the “GPCR A *family*-*near*” conformation (Fig. 4(a)). An analysis of the J_i_-scores for individual dihedral angles (Fig. 4(b) and (c)) showed that most of the scores were also in the range of “GPCR A *family*-*like*” or “GPCR A *family*-*near*”. These results indicate that the 20 computational models have a relatively close distance to the global and local conformations of the GPCR A proteins. Presumably, the conformations of the modeled structures mostly resemble the native GPCR structures because the experimental restraints were used in the computational modeling of the structures^54,55^. On the other hand, some J_i_-scores of 7 computational models were found in the range of “GPCR A *family*-*far*” (Ω_1_ of *Olfactory receptor 5AC1*, Ω_2_ and λ_1_ of *Gastric inhibitory polypeptide receptor*, Ω_2_, Ω_3_, and Ω_5_ of *Neuropeptide FF receptor 2*, Ω_2_ of *Neuromedin*-*K receptor*, Ω_2_ of *Olfactory receptor*, λ_1_ of *Glucagon*-*like peptide 2 receptor*, and λ_4_ of *GPCR 2 Secretin*-*like receptor*). This indicates that the local conformations related to the dihedral angles in the modeled structures somewhat deviate from the native 27 GPCR structures. To check whether the templates used in the GPCR I-TASSER modeling are related to these local deviations, 24 templates of GPCR structures used in the modeling were validated by estimating their J-scores against our 27 GPCR dataset. It was observed that all of the 24 templates showed J-_tot_ and J_i_-scores of *“family*-*like”* or “*family*-*near*” range, and there were no templates showing J-scores of “*family*-*far*” (data not shown). Therefore, at least the 24 templates used in the GPCR I-TASSER modeling might not induce the J_i_-scores of “*family*-*far*” in the 7 models. It is presumed that the local deviations of the models are induced in the next modeling steps such as threading, *ab initio* modeling, and energy minimization.

In summary, how much the computational models reproduce the native conformation of GPCR A proteins could be estimated at global and local conformational level. None of the validated 20 models deviated significantly from the native GPCR protein in terms of the global conformation, but some models showed locally different conformations. The deviated local angles in some models can be interpreted in two ways. One is that the computational models are correct and their real structures have the dihedral angles with a deviation from those of 27 native structures. The other is that the modeling of the local structure may not be correct. Of course, this may not be confirmed before their structures are experimentally identified.

### Measurement of conformational difference between active GPCR and inactive GPCR

In general, the activation of GPCR proteins is triggered by the binding of diverse ligands. The binding induces conformational changes in the GPCR proteins specific to the receptor types, which in turn activates the associated G protein. This eventually leads to modulation of various intercellular signaling pathways and changes in the downstream canonical cellular biochemistry. Understanding the conformational changes in the GPCR proteins from an inactive state to active state is crucial in receptor-ligand interactions and the subsequent signal pathways. Many studies have been performed at the molecular level, which provided useful information on the changes in the TM helical interactions in the activation^20,33,56,57^. In this study, an attempt was made to measure the global and local conformational distance of activated and inactivated GPCR proteins by comparing their J-scores to understand their conformational change at the macroscopic joint-based dihedral level.

**Figure 5:**
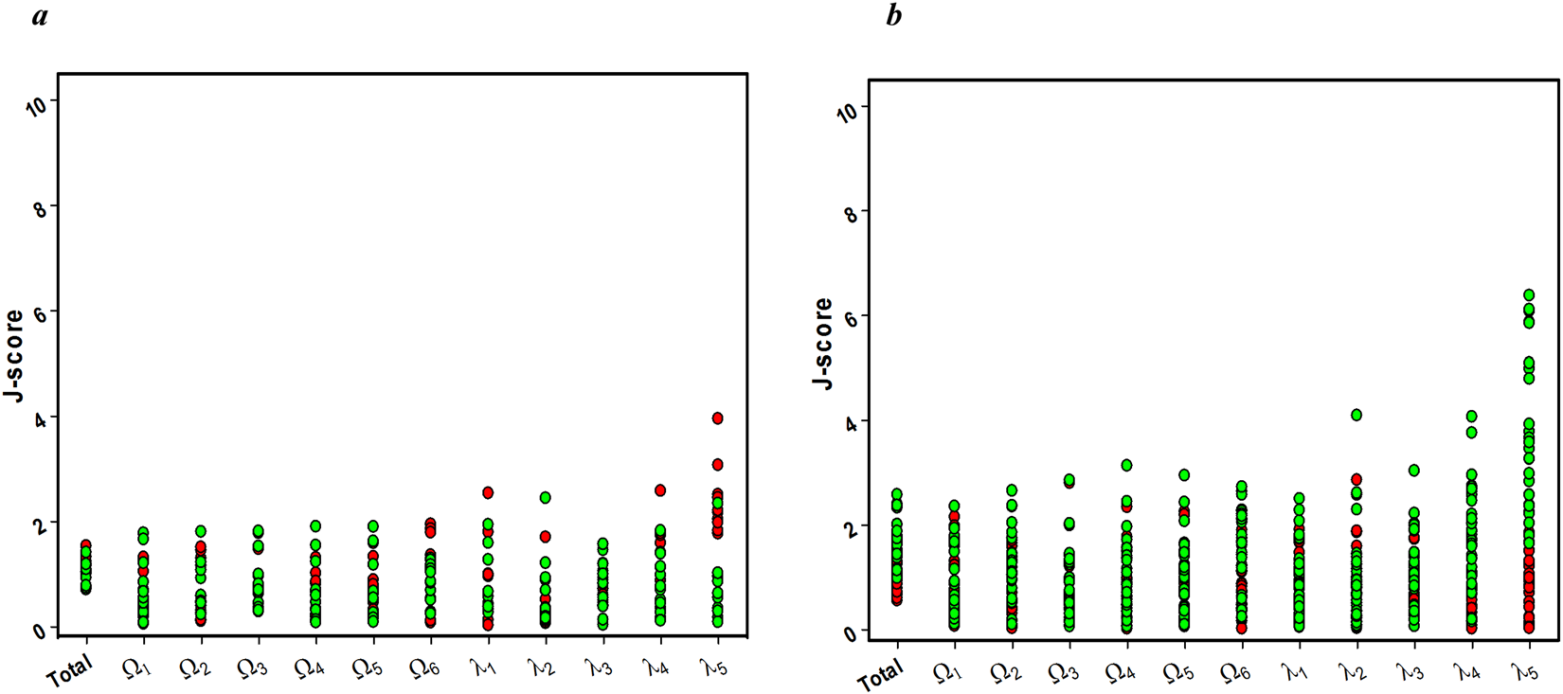
J-scores of active states or inactive states against active reference and inactive reference. (**a**) J-scores of 10 active structures against the inactive reference (red) and the active reference (green), and (**b**) J-scores of 27 inactive structures against the inactive reference (red) and the active reference (green).

To study the conformational distance between inactive states and active states, the dataset for active states were constructed by selecting 10 non-redundant active-like structures (*4UHRA: Adenosine receptor A2a*, *3SN6R: β2 adrenergic receptor*, *5GLHA: Endothelin B receptor*, *4MQSA: Muscarinic acetylcholine receptor*, *4GRVA: Neurotensin receptor type 1*, *4PXFA: Opsin receptor*, *4X1H: Bovine rhodopsin*, *4XT1A: Viral GPCR*, *5C1MA: Opioid mu receptor*, *and 4IB4A: 5*-*hydroxytryptamine receptor*) from all the available active-like state structures of Class A GPCR in PDB. Dihedral angles of the active-like conformations were calculated and tabulated in SI Table 9. First, the J-scores of the 10 active states were estimated by using the scoring function devised on the basis of the initial 27 inactive dataset as a reference (SI Table 1), which indicates the distance of each active state from the average of inactive conformation. As a control, J-scores of the 10 active states were also calculated using the scoring function devised on the basis of the 10 active states as a reference dataset, which indicates the distance of each active state from the average of active conformation. As shown in Fig. 5(a), the 10 active structures against inactive reference set showed slightly but clearly higher values than the control in the J_i_-score for λ_5_, whereas their other J-scores were almost similar to those of the control. Second, the analyses were replicated with the 27 inactive GPCR proteins against the active reference and inactive reference, leading to the almost same pattern (Fig. 5(b)). These results imply that there is a marginal but a clear conformational difference between the active and inactive states, related to the local λ_5_ dihedral angle of the joint-based coordinate.

Overall, the joint-based macroscopic descriptor with the J-score measurement could be used to detect the conformational distance between the active and inactive state structures of GPCR at the macroscopic level. The most distant dihedral angle between the two states was λ_5_. From this finding, activation of the GPCR by ligand-binding is believed to cause the local conformational change, particularly related to the λ_5_ dihedral angle. In the ***Discussion*** section, an attempt is made to interpret the conformational change in GPCR by relating the λ_5_ dihedral angle variation to the TM helical arrangement and extension pattern in the GPCR protein.

## Discussion

The joint-based descriptor was applied to quantify the conformational distance of the 7TM proteins from the GPCR A family, to examine the conformational difference between the active and inactive states of GPCR, and to validate the GPCR computational models. A prominent feature of the approach is to measure the structural distance at the macroscopic level, which permits an analysis of the conformational difference of complex proteins, such as TM proteins, in a more simplistic way. This study focused on GPCR proteins and their related structures, but the approach can also explore the geometrical similarities and diversities that are particular to any TM topology. The structural features, evolutionary relationships, computational models, and conformational changes of TM proteins can be studied in a more effective way if the joint-based approach is combined with the microscopic approaches that are popularly utilized for measuring the structural difference.

In general, the more the protein structural descriptor is macroscopic, the more the local microscopic information about protein structure is lost. The joint-based descriptor is a macroscopic one that employs only the dihedral angles of joints of secondary structures as a coordinate, and therefore it cannot detect many important local structural features of TM structures such as helical bending or kinks, interhelical contacts, loop variations, and the tilt of the first and the last helices. These local features can be efficiently captured through more microscopic approach such as RMSD of C-alpha atoms. Therefore, it should be noted that there may be no direct correlation between *Cα*-*based RMSD* and the joint-based distance. Despite the limitation of joint-based approach in the detection of microscopic structural features, the use of the joint-based approach might be meaningful in the aspect that protein topology can be studied in a new viewpoint, using the dihedral angles of the joints of secondary structures as structural coordinate, which was not previously explored. It is expected that the joint-based approach can be a tool to study protein structures together with existing approaches.

As reported in our previous study^38^, dihedral angles between the joints can be roughly related to the arrangement and extension patterns of the TM helices in the membrane proteins at the macroscopic level. Briefly, the bending and kinked angles of most TM helices are known to be comparatively low (less than 20 degrees)^58^, and the TM helices are assumed to be straight lines of the joint points, as shown in SI Fig. 1. The Ω_i_ dihedral angle represents how the i+1^th^ TM helix (H_i+1_) is arranged or tilted against the i^th^ TM helix (H_i_). The λ_i_ dihedral angle provides information on how the TM helices H_i_, H_i+1_, and H_i+2_ are extended or packed. Most helices in TM proteins are relatively parallel and therefore the relative position of the four joint points for λ_i_ can be roughly related to the extension of the three continuous helices. Then, the local distances measured in the conformational study of the GPCR A family proteins and the other 7TM proteins can be related to their helical arrangement/extension patterns. For example, the proteins in the microbial and algal rhodopsin family showed much higher J_i_-scores for λ_1_ and λ_3_ than the other dihedral angles. This suggests that the family has a very different conformation from the GPCR A family in the extension patterns of H_1_, H_2_, & H_3_ and H_3_, H_4_, & H_6_. Another example is that the Ω_4_ dihedral angle is a relatively conserved dihedral angle in the entire 7TM proteins analyzed, which suggests that the 7TM proteins have a relatively similar local conformation in terms of the helical arrangement of H_5_ against H_4_, compared to the other helical arrangement and extension patterns.

In the study on the validation of computational models, we attempted to check how much the computational models of GPCR proteins that were already validated in many aspects are close to the native GPCRs only at the level of the joint-based coordinate. However, it should be noted that the joint-based validation alone cannot be used to validate the computational models properly in the validation of raw computational models, because, as mentioned above, the joint-based descriptor cannot detect many important local structural features of TM structures. It should be used together with other microscopic validation tools which can detect other structural features such as interhelical contacts of TM proteins. The joint-based approach is expected to be an additional tool that can validate the conformational topology of computational models.

**Figure 6:**
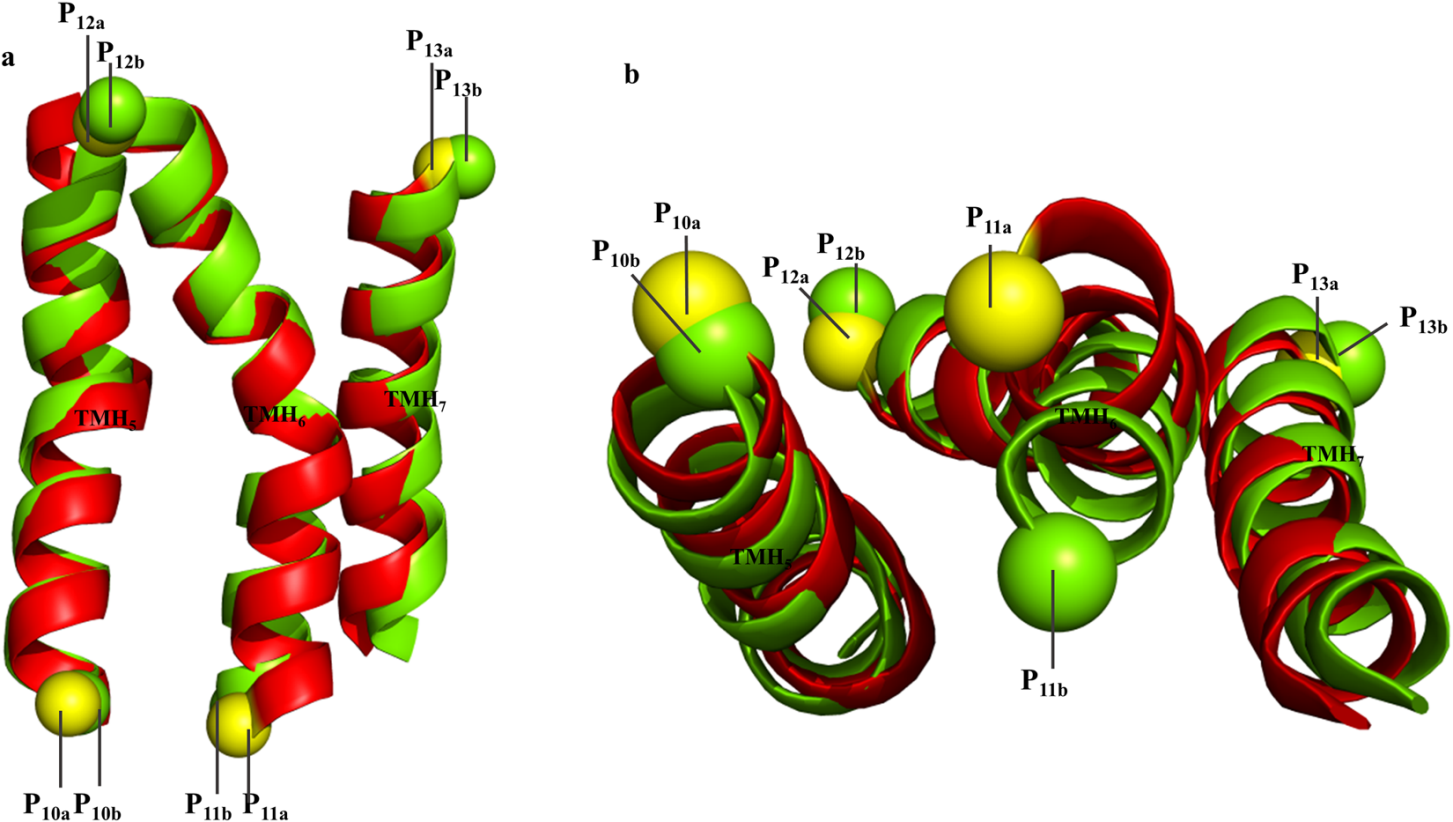
Comparison of conformational difference between inactive (red) and active-like (green) GPCR_A structures. (**a**) Side view of the linearly ordered TMH_5_, TMH_6_, and TMH_7_ helices of GPCR structures. P_10a_ and P_10b_ are the joint points belong to the cytoplasmic ends of TM_5_ of inactive and active structures, respectively. TMH_6_ has P_11a_ and P_11b_; P_12a_ and P_12b_ and P_13a_ and P_13b_ belong to extracellular ends of TMH_7_. (**b**) Top view of the arrangement of three consecutive helices TMH_5_-TMH_6_-TMH_7_. GPCR activation causes the macroscopic transition at the cytoplasmic end of TMH_6_ towards TMH_7_ and induces the rearrangement of P_11a_ to P_11b_, which leads to the change of λ_5_. The figures present the example of inactive [2RH1]_a_ and active [3SN6]_b_ pairs.

In the study on the conformational distance between the inactive and active GPCR proteins, λ_5_ was identified as the major dihedral angle that was most commonly and prominently changed. Based on the relationship between the dihedral angle type and the arrangement or extension pattern of the TM helices, the change in the λ_5_ dihedral angle in the GPCR conformation shows that there is a conformational change in the extension pattern in H_5_, H_6_, and H_7_. This conformational change is consistent with previous reports showing that the cytoplasmic ends of H_6_ and H_7_ in GPCR regularly incline to be tilted from the helix bundle during the receptor-ligand interactions^33,59–63^. To better understand the conformational change related to GPCR activation, the λ_5_ dihedral angles of the active and inactive states were compared directly, and their geometrical relationship with the extension pattern of H_5_, H_6_, and H_7_ was analyzed further. The λ_5_ angles of the inactive and active states of the ten pairs were identified to be in the range of −116° to −173°, and +133° to +177°, respectively. These values suggest that the conformational change by activation is consistent and somewhat symmetrical. λ_5_ is defined based on the four joint points (P_10_, P_11_, P_12_, and P_13_) in H_5_, H_6_, and H_7_ of Fig. 1. Therefore, this study examined whether there is real symmetry and what causes the symmetrical conformational change by comparing the arrangement of joint points in H_5_, H_6_, and H_7_ in the PDB structures. The cytoplasmic end of TM_6_ was bent slightly toward the TM_7_ by activation, leading to symmetrical variations of the helical extension pattern. Figure 6 presents an example of the identified symmetrical difference in the active-inactive pairs at the joint coordinate level.

## Methods

### Datasets used in the study

All the proteins analyzed in this study were selected from PDB and high-resolution (<3.5 Å) structures. The dataset of 27 representative GPCR-A family proteins were achieved as follows. First, all the x-ray crystal structures belonging to GPCR_A family, and 155 monomeric chain structures were found. Subsequently, 27 chains of inactive states were filtered as a non-redundant dataset by selecting all the available different subfamily receptor types. The dataset for 10 active states was prepared by selecting non-redundant proteins showing different subfamily receptors from 32 structures annotated as active-like conformations in PDB. To obtain proteins that represent four different families in the rhodopsin-like superfamily, the proteins in microbial and algal rhodopsin, Class B (Secretin), Class C (Glutamate), and Class F (Frizzled), were collected and the non-redundant sequences were extracted. The proteins for 12 superfamilies (Bacterial Cytochrome C Oxidase, Methane Monooxygenase, Maltose Transporters, Zinc Metalloprotease, Human γ secretase, Glutamate Ion Channel, Protein YetJ, Metal Transport, Prolipoprotein Diacylglyceryl transferase, Sweet Transporter and Cation Channel proteins) were also selected based on their sequence redundancy. A dataset of 20 computational models was isolated from the GPCR-HGmod database, which is the library of human GPCR-predicted models generated through GPCR I-TASSER^54^. Approximately 1000 GPCR models are publicly available to download from http://zhanglab.ccmb.med.umich.edu/GPCR-HGmod/ and are assigned by unique HG ID and UniProt ID. There are 1 to 5 models for each entry, which are assisted by the TM-score and RMSD values. They have also been assigned a confidence score for each top model, which ranges between the values −5 to 2; a higher score indicates the quality of the model. Ten high TM-score models [*P08100*, *Q0PJU0*, *Q14332*, *P48146*, *Q6IEZ2*, *P21731*, *P58182*, *P59536*, *P0C628*, *and Q5CZ62*], and 10 low TM-score models [*B9EIL6*, *P21453*, *P48546*, *Q9Y5X5*, *P29371*, *P50406*, *O95838*, *P35348*, *Q6ZMH4*, *and P28223*] were selected randomly.

### Joint-based representation and Ω/λ dihedral measurements

The beginning and ending residue Cα atoms of the TM segment were projected as a joint coordinate for the dihedral calculation, as described elaborately in a previous report^38^. Selection of the structural joints was scrutinized visually for the Cα XYZ coordinates from the corresponding PDB file. OPM was referred to define the helix boundary and TM segments for the crystal structures^64^. In addition, for all the selected sequences and predicted models, their TM boundaries were defined by the membrane topology prediction tool called the TOPCONS suite^65^. While establishing a connection of the joint residues, a new description of the overall protein structure was portrayed. The developed program parses the query structures and the Cα XYZ coordinates preselected from each joint were exploited for the dihedral measurements, as described previously. The resulting number of dihedral angles for each protein is directly proportional to the number of helices and loops present in them. The compiled data set was used for the dihedral angle measurements by the joint based approach and used for the structural diversity assessments.

## Electronic supplementary material

Supplementary Information
