# Supplementary material for: Measuring the Conformational Distance of GPCR-related Proteins Using a Joint-based Descriptor
Source: Sci Rep. 2017 Nov 9;7:15205. doi: 10.1038/s41598-017-15513-3 (PMC5680341; doi:10.1038/s41598-017-15513-3)

Supplementary Information

Measuring the Conformational Distance of GPCR-related Proteins Using a Joint-based Descriptor

Jayaraman Thangappan[1], Bharat Madan[1], Sangwook Wu[2]* and, Sun-Gu Lee[1]*

[1]Department of Chemical Engineering, Pusan National University, Busan, 609-735, Republic of Korea

[2]Department of Physics, Pukyong National University, Busan, 608-737, Republic of Korea

Corresponding Authors:

Sangwook Wu

Department of Physics, Pukyong National University, Busan, 608-737, Republic of Korea

Telephone: +82-51-629-5578

E-mail: sangwoow@pknu.ac.kr

Sun-Gu Lee

Department of Chemical Engineering, Pusan National University, Busan, 609-735, Republic of Korea

Telephone: +82-51-510-2786

E-mail: sungulee@pusan.ac.kr

Table S1: PDB IDs and dihedral angles of non-redundant Class A GPCR structures.

#	PDB	Ω_1	Ω_2	Ω_3	Ω_4	Ω_5	Ω_6	λ_1	λ_2	λ_3	λ_4	λ_5	Subfamily
1	4IARA	-11.33	-15.22	19.92	-15.79	-18.82	-9.14	-86.98	131.66	5.69	-60.08	-165.29	5-hydroxytryptamine receptor 1B
2	5IU4A	-13.15	-15.99	20.00	20.22	-14.44	-2.06	-127.36	137.17	-18.16	-83.72	-163.38	Adenosine receptor A2
3	5VBLB	-14.00	-21.60	17.67	-17.50	-25.65	2.92	-62.82	162.95	51.05	-34.03	-165.71	Apelin receptor
4	4BVNA	-9.02	-22.65	11.80	-7.10	-13.03	-2.48	-118.05	112.89	-5.46	-43.67	-163.63	β1 adrenergic receptor
5	2RH1A	-17.23	-22.42	19.92	-9.00	-20.12	1.15	-121.20	109.13	-0.83	-35.58	-151.52	β2 adrenergic receptor
6	4MBSA	-12.70	-17.31	14.31	-12.68	-22.00	-3.25	-104.45	127.32	24.10	-42.15	-171.35	Chemokine
7	5TGZA	-22.48	-12.98	10.96	-11.46	-16.06	-12.45	-134.69	73.27	-14.16	-55.55	-144.43	Cannabinoid receptor
8	3PBLA	-13.13	-16.20	14.84	-5.33	-24.33	1.27	-109.55	127.34	-14.77	-28.98	-109.40	Dopamine
9	5GLIA	-13.58	-11.95	14.57	-16.76	-29.64	3.73	-93.20	137.36	26.95	-39.24	-160.75	Endothelin
10	4PHUA	-7.83	-16.03	28.97	12.89	-18.44	-18.08	-91.83	163.35	35.26	-44.42	-160.88	Free fatty acid receptor
11	3RZEA	-16.05	-21.08	22.39	7.73	-14.04	-1.72	-98.95	134.31	-31.90	-22.22	-116.30	Histamine
12	4Z35A	-14.15	-21.18	17.23	-0.88	-23.40	-3.78	-133.92	108.87	-14.21	-66.33	-157.34	Lysophosphatidic
13	5CXVA	-13.81	-17.60	18.71	5.05	-17.23	-4.88	-96.93	143.07	32.86	-36.38	-171.24	Muscarinic (Acetylcholine)
14	4BUOA	-11.49	-13.44	14.55	-9.64	-6.31	0.20	-78.84	126.69	1.16	-23.36	-116.51	Neurotensin
15	4EA3A	-19.41	-21.09	13.10	-1.63	-11.97	0.37	-78.54	121.02	-4.82	-24.18	-113.49	Nociceptin/Orphanin
16	4N6HA	-5.56	-13.58	12.88	-8.25	-12.09	-1.39	-71.71	123.81	-2.32	-4.75	-128.24	Opioid delta receptor
17	4DJHA	-19.39	-9.88	17.56	-22.36	-22.36	1.14	-67.34	144.92	37.11	-25.60	-148.94	Opioid kappa receptor
18	4DKLA	-15.28	-21.56	12.92	1.22	-17.38	-9.09	-97.66	125.06	-5.39	-46.23	-130.78	Opioid mu receptor
19	4S0VA	-16.19	-16.14	15.37	-29.04	-16.21	0.85	-88.83	137.30	25.15	-8.80	-113.09	Orexin receptor
20	4XNVA	-15.66	-11.58	11.41	-29.70	-19.62	-13.28	-111.51	139.72	51.41	-32.97	-151.41	Purinoreceptor
21	1U19A	-13.09	-15.92	25.48	-18.66	-24.02	-2.21	-101.67	115.73	18.33	-31.28	-155.30	Rhodopsin
22	2Z73A	-13.74	-14.17	20.82	1.73	-20.11	-0.02	-108.73	146.87	16.27	-30.09	-142.54	Squid rhodopsin
23	3V2YA	-22.49	-8.84	18.90	-3.92	-12.78	0.34	-113.93	111.08	6.26	-55.05	-146.20	Sphingosine
24	3VW7A	-11.27	-9.83	19.27	-21.82	-13.95	-14.82	-77.37	140.84	26.47	-19.28	-147.23	Thrombin (Proteinase-activated I)
25	5NDDA	-21.48	-14.62	13.91	-27.24	-10.13	-23.29	-119.47	117.20	34.73	-40.04	-168.11	Thrombin (Proteinase-activated II)
26	4ZUDA	-12.34	-10.94	14.93	-22.19	-17.57	-8.10	-103.80	132.06	33.16	-22.74	-126.78	Type-1 angiotensin II receptor
27	4XT3A	-12.71	-14.24	15.76	-20.62	-19.46	-12.63	-123.81	97.52	24.99	-36.94	-173.33	Viral GPCR US28

Table S2: **J-scores of overall, Ω-type and λ type dihedral angles for Class A GPCR family structures**

Protein family	***J-score***													
	#	***PDB***	***Total***	Ω_1	Ω_2	Ω_3	Ω_4	Ω_5	Ω_6	λ_1	λ_2	λ_3	λ_4	λ_5
GPCR_A	1	4IARA	0.7	0.7	0.2	0.7	0.5	0.2	0.6	0.7	0.2	0.3	1.4	0.9
	2	5IU4A	1.3	0.3	0.0	0.7	2.3	0.6	0.4	1.3	0.5	1.4	2.7	0.8
	3	5VBLB	1.2	0.1	1.4	0.2	0.6	1.5	1.1	1.9	1.9	1.7	0.2	0.9
	4	4BVNA	0.9	1.3	1.6	1.2	0.2	0.9	0.3	0.9	0.8	0.8	0.4	0.8
	5	2RH1A	0.8	0.7	1.6	0.7	0.1	0.4	0.9	1.0	1.0	0.6	0.1	0.2
	6	4MBSA	0.5	0.4	0.4	0.6	0.2	0.8	0.2	0.2	0.0	0.5	0.3	1.2
	7	5TGZA	1.4	2.0	0.7	1.4	0.1	0.3	1.1	1.7	2.9	1.2	1.1	0.1
	8	3PBLA	0.9	0.3	0.1	0.5	0.3	1.2	0.9	0.4	0.0	1.2	0.5	1.8
	9	5GLIA	0.9	0.2	0.9	0.6	0.6	2.2	1.2	0.4	0.5	0.6	0.1	0.7
	10	4PHUA	1.4	1.6	0.0	2.8	1.8	0.1	1.9	0.5	1.9	1.0	0.4	0.7
	11	3RZEA	1.1	0.4	1.3	1.3	1.4	0.7	0.5	0.1	0.4	2.0	0.9	1.5
	12	4Z35A	1.0	0.1	1.3	0.1	0.7	1.1	0.2	1.7	1.0	1.2	1.7	0.5
	13	5CXVA	0.7	0.1	0.4	0.4	1.2	0.1	0.0	0.2	0.8	0.9	0.0	1.2
	14	4BUOA	1.0	0.7	0.6	0.6	0.0	2.2	0.7	1.1	0.1	0.5	0.8	1.5
	15	4EA3A	1.0	1.2	1.3	0.9	0.6	1.1	0.7	1.1	0.4	0.8	0.7	1.6
	16	4N6HA	1.1	2.1	0.6	1.0	0.1	1.1	0.5	1.5	0.2	0.7	1.9	0.9
	17	4DJHA	1.0	1.2	1.4	0.1	1.0	0.9	0.9	1.7	0.9	1.1	0.7	0.1
	18	4DKLA	0.7	0.2	1.4	0.9	0.9	0.1	0.6	0.2	0.1	0.8	0.6	0.8
	19	4S0VA	0.9	0.4	0.1	0.4	1.5	0.3	0.8	0.6	0.5	0.6	1.6	1.7
	20	4XNVA	1.0	0.3	1.0	1.3	1.6	0.3	1.2	0.5	0.6	1.7	0.2	0.2
	21	1U19A	0.8	0.3	0.0	2.0	0.7	1.2	0.4	0.0	0.6	0.3	0.3	0.4
	22	2Z73A	0.6	0.2	0.4	0.9	0.9	0.4	0.7	0.4	1.0	0.2	0.4	0.2
	23	3V2YA	1.0	2.0	1.7	0.5	0.5	1.0	0.7	0.7	0.9	0.3	1.1	0.0
	24	3VW7A	0.9	0.8	1.5	0.5	0.9	0.7	1.4	1.2	0.7	0.6	1.0	0.0
	25	5NDDA	1.3	1.7	0.3	0.7	1.4	1.5	2.6	0.9	0.6	1.0	0.2	1.1
	26	4ZUDA	0.7	0.5	1.2	0.5	1.0	0.1	0.5	0.2	0.2	0.9	0.8	1.0
	27	4XT3A	0.9	0.4	0.4	0.2	0.9	0.3	1.1	1.2	1.6	0.6	0.0	1.3

Table S3: PDB IDs and dihedral angles of rhodopsin-like superfamily structures.

#	PDB	Ω_1	Ω2	Ω_3	Ω_4	Ω_5	Ω_6	λ_1	λ_2	λ_3	λ_4	λ_5	Family
1	5G2CA	-22.01	1.19	4.78	-9.95	1.94	-11.77	152.28	129.82	108.63	-65.62	167.89	
2	5G28A	-21.86	1.73	4.45	-9.57	2.4	-11.57	151.8	129.16	108.69	-67.15	166.2	
3	4XTLA	-17.87	-6.12	2.41	-18.95	-9.33	-19.17	176.18	179.65	114.31	-103.53	110.59	
4	1M0KA	-21.58	-4.9	10.68	-10.82	-6.26	-12.2	175.8	141.59	97.56	-59.29	152.27	
5	4HYJA	-19.63	-4.35	14.56	-25.3	-16.2	-1.08	150.85	116.89	101.14	-26.61	-171.54	
6	5JJEB	-20.21	-18.21	14.5	-24.63	-7.58	-4.26	154.68	138.02	111.21	-21.78	-156.52	
7	3QAPA	-23.39	-12.73	13.33	-17.48	-0.28	2.56	176.93	145.2	102.48	-87.56	111.18	Microbial and Algal rhodopsin
8	3DDLA	-17.91	-21.4	20.56	-23.95	-11.78	-19.48	168.12	150.84	106.82	-42.55	173.94	
9	1XIOA	-18.54	-9.46	12.59	-21.95	-13.91	-2.69	144.61	136.65	100.22	-46.13	143.59	
10	5B0WA	-23.2	-5.53	9.07	-24.03	-12.32	-6.33	137.67	124.24	97.73	-63.79	130.16	
11	5G2DA	-22.17	1.41	4.72	-9.52	2.2	-11.81	152.7	129.21	107.82	-67.09	167.34	
12	5AX0A	-18.63	-5.62	8.46	-21.51	-5.52	-5.09	162.28	144.95	123.66	-33.56	176.95	
13	3UG9A	-30.18	-8.37	12.19	-22.55	2.7	-22.21	160.22	152.66	131.6	-47.05	164.92	
1	4Z9GA	-27.68	-22.46	15.98	9.94	-26.21	-11.17	-89.05	127.73	-4.96	-62.58	-137.37	
2	4K5YA	-23.09	-20.82	14.93	20.31	-25.52	-12.15	-80.87	117.45	-22.04	-25.36	-92.02	
3	4L6RA	-27.23	-18.86	36.39	-12.38	-27.81	-1.23	-103.47	145.92	16.83	-33.76	-159.43	Class B (Secretin)
4	5EE7A	-23.11	-19	24.25	17.12	-15.8	-10.33	-102.57	117.95	-15.68	-77.13	-124.21	
5	5VEWA	-29.79	-13.06	22.91	-18.51	-20.56	-14.79	-133.53	121.23	20.77	-42.30	-136.86	
1	5CGDA	-9.74	-29.3	27.76	12.47	-18.9	14.63	-120.44	128.42	-34.11	-100.97	114.95	
2	5CGCA	-9.72	-29.19	28.02	12.23	-19.08	14.57	-121.25	129.22	-33.29	-101.12	114.8	Class C (Glutamate)
3	4OO9A	-10.11	-29.34	19.25	-26.74	-18.49	14.51	-128.46	133.83	-22.26	-90.16	158.86	
4	4OR2A	-8.78	-24.82	26.64	16.24	-13.33	1.23	-113.94	134.62	-26.85	-105.07	150.47	
1	4JKVA	-15.75	-19.6	28.76	13.46	-11.82	-15.31	-100.11	158.45	-33.82	-82.62	-147.84	
2	5L7DA	-14.62	-15.79	32.37	13.84	-16.08	-16.55	-100.55	136.77	-0.44	-77.07	-132	
3	5L7IA	-11.17	-18.35	31.94	4.39	-16.33	-12.17	-103.33	129.84	1.31	-67.82	-149.85	
4	4O9RA	-16.33	-22.44	35.23	2.15	-20.87	-13.01	-117.39	142.38	12.09	-62.29	-147.08	Class F (Frizzled)
5	4QINA	-11.13	-15.82	20.35	2	-19.42	-9.37	-118.99	132.5	22	-76.05	-166.84	
6	4N4WA	-17.79	-14.15	32.17	11.67	-18.28	-7.04	-109.63	133.01	-6.47	-65.19	-154.51	
7	4QIMA	-17.29	-22.24	36.11	4.21	-18.39	-12.88	-119.55	135.83	7.95	-64.65	-149.62	

Table S4: **J-scores of overall, Ω and λ type dihedral angles for different families in rhodopsin-like superfamily**

Protein families	*J-score*													
	**#**	**PDB**	***Total***	Ω_1	Ω_2	Ω_3	Ω_4	Ω_5	Ω_6	λ_1	λ_2	λ_3	λ_4	λ_5
Microbial and Algal rhodopsin	1	5G2CA	3.0	1.9	4.1	2.8	0.0	3.8	1.0	5.3	0.1	4.3	1.7	2.2
	2	5G28A	3.0	1.8	4.2	2.9	0.0	3.8	1.0	5.4	0.1	4.3	1.8	2.3
	3	4XTLA	3.2	0.8	2.3	3.4	0.7	1.6	2.1	4.1	2.7	4.6	3.9	5.0
	4	1M0KA	2.4	1.7	2.6	1.5	0.1	2.2	1.1	4.2	0.7	3.8	1.3	3.0
	5	4HYJA	2.3	1.3	2.8	0.6	1.2	0.3	0.5	5.4	0.6	4.0	0.6	1.2
	6	5JJEB	2.3	1.4	0.6	0.6	1.2	1.9	0.1	5.2	0.5	4.4	0.9	0.5
	7	3QAPA	2.8	2.2	0.8	0.8	0.6	3.3	1.1	4.1	0.9	4.0	3.0	5.0
	8	3DDLA	2.2	0.9	1.3	0.8	1.1	1.2	2.1	4.5	1.2	4.2	0.3	1.9
	9	1XIOA	2.5	1.0	1.5	1.0	1.0	0.7	0.3	5.7	0.5	3.9	0.5	3.4
	10	5B0WA	2.8	2.1	2.5	1.8	1.1	1.0	0.2	6.1	0.2	3.8	1.6	4.1
	11	5G2DA	3.0	1.9	4.2	2.8	0.0	3.8	1.0	5.3	0.1	4.3	1.8	2.3
	12	5AX0A	2.5	1.0	2.5	2.0	0.9	2.3	0.0	4.8	0.9	5.0	0.2	1.8
	13	3UG9A	3.0	3.8	1.8	1.1	1.0	3.9	2.5	4.9	1.3	5.3	0.6	2.4
Class B1 (Secretin)	1	4Z9G	1.4	3.2	1.6	0.2	1.5	1.6	0.9	0.6	0.0	0.8	1.5	0.5
	2	4K5Y	1.5	2.1	1.2	0.5	2.3	1.5	1.0	1.0	0.5	1.6	0.7	2.7
	3	4L6R	1.8	3.1	0.7	4.5	0.2	1.9	0.5	0.1	1.0	0.2	0.2	0.6
	4	5EE7	1.4	2.1	0.8	1.7	2.1	0.3	0.8	0.1	0.5	1.3	2.3	1.1
	5	5VEW	1.4	3.7	0.7	1.4	0.7	0.6	1.4	1.6	0.3	0.4	0.3	0.5
Class C (Glutamate)	1	5CGD	2.6	1.1	3.2	2.5	1.7	0.3	2.8	1.0	0.0	2.1	3.7	4.8
	2	5CGC	2.6	1.1	3.2	2.6	1.7	0.3	2.8	1.0	0.1	2.1	3.7	4.8
	3	4OO9	2.0	1.0	3.3	0.5	1.3	0.2	2.8	1.4	0.3	1.6	3.1	2.7
	4	4OR2	2.1	1.4	2.2	2.2	2.0	0.8	0.9	0.7	0.4	1.8	4.0	3.1
Class F (Frizzled)	1	4JKV	1.6	0.3	0.9	2.7	1.8	1.1	1.5	0.0	1.6	2.1	2.7	0.1
	2	5L7D	1.5	0.1	0.0	3.6	1.8	0.3	1.7	0.0	0.5	0.6	2.3	0.7
	3	5L7I	1.3	0.8	0.6	3.5	1.1	0.2	1.1	0.1	0.1	0.5	1.8	0.2
	4	4O9R	1.6	0.5	1.6	4.2	0.9	0.6	1.2	0.8	0.8	0.0	1.5	0.0
	5	4QIN	1.0	0.8	0.0	0.8	0.9	0.4	0.7	0.9	0.3	0.4	2.3	1.0
	6	4N4W	1.4	0.8	0.4	3.5	1.7	0.1	0.3	0.4	0.3	0.9	1.7	0.4
	7	4QIM	1.6	0.7	1.5	4.4	1.1	0.2	1.2	0.9	0.4	0.2	1.6	0.1

Table S5: PDB IDs and dihedral angles of available superfamilies structures in the 7TM fold (Non-GPCR).

#	PDB	Ω_1	Ω2	Ω_3	Ω_4	Ω_5	Ω_6	λ_1	λ_2	λ_3	λ_4	λ_5	Superfamily
1	5LXG	-12.04	-24.89	10.82	-16.30	-28.13	6.53	-155.87	143.24	77.81	-47.58	116.43	Adiponectin Receptor
2	5LX9	-16.19	-15.95	6.29	3.13	-15.76	0.79	-161.00	-170.60	69.22	-106.50	110.62	
3	3WXV	-17.33	-16.36	9.24	3.71	-14.39	29.51	-154.40	-173.97	65.71	-102.42	110.00	
4	3WXW	-15.95	-17.57	7.06	2.15	-19.83	2.58	-166.66	-170.76	70.91	-109.99	111.99	
5	5LXA	-16.92	-15.59	6.97	3.03	-16.65	1.66	-159.67	-173.53	67.88	-106.69	110.25	
6	5LWY	-15.93	-16.88	6.27	2.41	-15.80	0.86	-165.40	-171.19	69.58	-104.19	112.00	
1	1QLE	-30.77	-16.74	6.48	-24.14	-15.46	-24.05	12.31	-80.71	113.44	-37.68	160.17	Bacterial Cytochrome c oxidase
2	1M56	-29.73	-22.54	-7.34	-17.8	-15.13	-36.48	3.79	-58.19	116.99	-57.3	141.27	
3	2DYR	-31.64	-32.45	-4.41	-17.78	-28.08	-20.95	24.85	-72.54	110.4	-19.41	159.26	
1	1YEW	21.11	124.14	-3.77	18.92	33.52	51.93	-47.51	171.83	57.22	-143.07	-53.03	Methane Monooxygenase
2	3CHX	31.99	99.32	4.46	12.75	37.32	37.69	-89.73	-150.81	65.38	-154.74	-36.87	
1	3FH6	21.55	17.21	-19.53	-36.84	33.5	-3.16	128.68	77.8	42.59	-132.41	154	Maltose Transporters
1	4AW6	17.63	14.55	13.46	23.7	22.97	16.31	81.5	125.06	61.22	-152.14	-1.84	Zinc Metalloprotease
2	2YPT	17.27	14.58	13.56	24.52	23.04	16.26	81.66	124.83	60.93	-150.61	-0.75	
3	4IL3	7.48	20.45	13.03	17.73	25.42	12.2	76.26	109.46	33.7	-177.24	-35.11	
1	4UIS	58.59	39.42	34.38	21.6	-21.4	-39.76	3.68	-133.7	-149.34	114.51	94.85	Human Gamma Secretase
2	5A63	16.57	27.23	43.8	23.74	-18.86	-23.84	43.67	-132	-127.2	124.62	75.92	
1	5KBT	-12.97	-6.14	11.86	-7.08	-19.08	-15.48	-8.14	65.24	-104.56	160.84	5.49	Glutamate-gated Ion Channel
1	4PGR	28.77	-12.03	14.3	2.03	-17.99	-23.17	-45.44	-86.02	54.04	48.15	-39.98	Protein YetJ
2	4TKQ	28.77	-12.02	13.91	2.07	-17.99	-23.15	-45.79	-85.74	54.3	48.52	-39.89	
3	4PGW	-2.6	-40	42.04	-4.55	-28.66	-24.37	45.12	-144.07	17.98	60.31	-21.51	
4	4PGS	-4.35	-40.27	44.95	0.45	-28.12	-23.16	49.2	-149.73	7.46	56.92	-26.34	
1	4M58	31.98	21.52	57.09	22.17	11.3	18.33	48.69	-133.14	174.48	-2.43	87.55	Metal Transporters
2	4M5B	24.33	16.55	53.7	22.72	10.97	18.45	49.41	-136.2	175.97	-5.69	85.91	
3	4M5C	24.53	15.99	54.33	22.52	10.56	18.34	50.18	-134.48	176.45	-4.55	85.59	
1	5AZB	36.9	31.33	19.09	-25.25	7.19	-19.57	15.52	-178.59	-123.99	92.66	-117.62	Prolipoprotein Diacylglyceryl transferase
2	5AZC	35.9	32.82	18.53	-25.54	7.06	-19.81	17.76	-178.63	-124.81	92.63	-121.79	
1	5CTG	-20.65	-22.25	-7.54	27.59	-20.43	-18.72	37.2	31.58	38.3	-129.01	1.42	Sweet Transporters
2	5CTH	-25.61	-22.61	-6.79	28.89	-20.55	-15.03	37.68	28.96	36.16	-130.66	1.2	
1	5EIK	54.51	12.18	-31.11	59.54	23.25	10.81	-105.9	100.42	-106.26	-69.54	113.21	Cation Channels
2	5EGI	55.67	27.92	-30.79	56.39	23.37	10.18	-111.41	97.13	-113.72	-108.88	103.54	
3	5H35	45.34	28.62	11.27	35.15	25.35	22.52	-137.83	19.17	-162.2	-119.39	52.19	
4	5H36	39.14	30.95	34.98	23.52	26.52	21.56	-152.26	-28.53	136.75	-126.63	39.29	
5	5WTR	43.11	28.52	11.58	34.93	26.42	23.12	-138.34	19.13	-161.44	-120.29	52.85	

Table S6: **J-scores of overall, Ω type and λ type dihedral angles for different superfamilies in the 7TM fold (Non-GPCR structures)**

Protein families	J-score													
	#	PDB	Total	Ω_1	Ω_2	Ω_3	Ω_4	Ω_5	Ω_6	λ_1	λ_2	λ_3	λ_4	λ_5
Adiponectin Receptor	1	5LXG	2.2	0.6	2.2	1.4	0.5	2.0	1.6	2.8	0.8	2.9	0.6	4.8
	2	5LX9	2.6	0.4	0.0	2.5	1.0	0.3	0.8	3.0	3.2	2.5	4.1	5.0
	3	3WXV	2.9	0.7	0.1	1.8	1.1	0.6	4.9	2.7	3.1	2.4	3.8	5.1
	4	3WXW	2.7	0.4	0.4	2.3	0.9	0.4	1.1	3.3	3.2	2.6	4.3	5.0
	5	5LXA	2.6	0.6	0.1	2.3	1.0	0.2	0.9	2.9	3.1	2.5	4.1	5.1
	6	5LWY	2.6	0.4	0.3	2.5	1.0	0.3	0.8	3.2	3.2	2.6	3.9	5.0
Bacterial Cytochrome C oxidase	1	1QLE	3.7	4.0	0.2	2.4	1.1	0.4	2.7	5.7	7.9	4.5	0.1	2.6
	2	1M56	4.4	3.7	1.6	5.6	0.6	0.5	4.5	5.2	9.1	4.7	1.2	3.5
	3	2DYR	4.3	4.2	4.0	5.0	0.6	2.0	2.3	6.3	8.4	4.4	1.0	2.7
Methane Monooxygenase	1	1YEW	11.6	8.6	33.7	4.8	2.2	9.6	8.1	2.7	2.3	2.0	6.2	4.6
	2	3CHX	10.2	11.3	27.7	2.9	1.8	10.3	6.1	0.6	4.3	2.4	6.9	5.4
Maltose Transporter	1	3FH6	6.0	8.7	8.0	8.5	2.1	9.6	0.2	6.5	2.6	1.3	5.6	2.9
Zinc Metalloprotease	1	4AW6	5.8	7.8	7.3	0.8	2.6	7.6	3.0	9.1	0.1	2.2	6.7	7.1
	2	2YPT	5.8	7.7	7.3	0.8	2.7	7.6	3.0	9.1	0.2	2.2	6.6	7.2
	3	4IL3	5.7	5.3	8.7	0.9	2.1	8.1	2.4	8.8	1.0	1.0	8.2	5.5
Human Gamma Secretase	1	4UIS	8.3	17.7	13.3	4.0	2.4	0.7	5.0	5.2	5.2	7.2	8.8	5.8
	2	5A63	6.5	7.5	10.4	6.2	2.6	0.2	2.7	7.2	5.3	6.3	9.4	6.8
Glutamate-gated Ion Channel	1	5KBT	4.4	0.3	2.3	1.2	0.2	0.3	1.5	4.6	3.3	5.2	9.4	7.5
Protein YetJ	1	4PGR	4.7	10.5	0.9	0.6	0.9	0.1	2.6	2.8	7.7	1.9	4.9	5.3
	2	4TKQ	4.7	10.5	0.9	0.7	0.9	0.1	2.6	2.8	7.7	1.9	5.0	5.3
	3	4PGW	4.6	2.9	5.8	5.8	0.4	2.1	2.8	7.3	4.6	0.2	5.7	6.2
	4	4PGS	4.6	2.4	5.9	6.5	0.8	2.0	2.6	7.5	4.3	0.2	5.5	5.9
Metal Transporters	1	4M58	6.9	11.3	9.0	9.3	2.5	5.4	3.3	7.5	5.2	7.2	2.0	6.2
	2	4M5B	6.4	9.4	7.8	8.5	2.5	5.4	3.3	7.5	5.0	7.3	1.8	6.3
	3	4M5C	6.4	9.4	7.7	8.7	2.5	5.3	3.3	7.5	5.1	7.3	1.9	6.3
Prolipoprotein Diacylglyceryl Transferase	1	5AZB	6.4	12.4	11.4	0.5	1.2	4.6	2.1	5.8	2.8	6.1	7.5	1.4
	2	5AZC	6.4	12.2	11.7	0.4	1.2	4.6	2.1	5.9	2.8	6.1	7.5	1.2
Sweet Transporters	1	5CTG	4.3	1.5	1.5	5.7	2.9	0.5	2.0	6.9	5.0	1.2	5.4	7.3
	2	5CTH	4.4	2.7	1.6	5.5	3.0	0.6	1.5	6.9	5.2	1.1	5.5	7.3
Cation Channel	1	5EIK	7.4	16.7	6.8	11.2	5.4	7.7	2.2	0.3	1.4	5.3	1.9	4.9
	2	5EGI	8.0	17.0	10.5	11.1	5.2	7.7	2.1	0.5	1.6	5.7	4.2	5.4
	3	5H35	7.4	14.5	10.7	1.3	3.5	8.0	3.9	1.9	5.7	7.8	4.8	7.9
	4	5H36	7.4	13.0	11.3	4.2	2.6	8.3	3.8	2.6	8.2	5.6	5.2	8.6
	5	5WTR	7.3	14.0	10.7	1.3	3.5	8.2	4.0	1.9	5.7	7.8	4.9	7.9

***Table S7: HGmod IDs and dihedral angles of GPCR computational models* obtained from HGmod database.**

#	HGmod ID	Ω_1	Ω2	Ω_3	Ω_4	Ω_5	Ω_6	λ_1	λ_2	λ_3	λ_4	λ_5
1	P08100	-19.1	-19.28	29.69	-2.72	-28.33	3.1	-138.39	106.87	-4.72	-39.34	-152.18
2	Q0PJU0	-17.77	-23.85	29.27	-2.28	-28.27	3.28	-122.48	127.67	-5.75	-39.63	-152.07
3	Q14332	-21.36	-1.89	26.92	-31.4	-21.2	-13	-145.13	105.47	10.69	-33.17	-151.93
4	P48146	-20.29	-27.48	7.65	-25.64	-13.81	-10.41	-160.8	132.11	31.11	-26.31	-138.46
5	Q6IEZ2	-17.84	-19.41	29.29	18.07	-14.75	2.52	-148.84	96.07	-27.65	-76.34	-162.81
6	P21731	-15.14	-17.91	17.45	-36.38	-21.52	3.95	-110.24	146.03	41.93	-20.42	-158.67
7	P58182	-28.31	-23.27	26.33	1.34	-26.33	10.48	-123.81	89.62	-15.05	-38.94	-163.1
8	P59536	-15.48	-24.11	17.05	-26.82	-17.78	-9.07	-156.04	120.35	10.04	-37.99	-147.84
9	P0C628	10.21	-22.24	18.12	-4.74	-24.59	3.1	-148.38	137.6	39.48	-67.48	-172.21
10	Q5CZ62	-19.66	-30.5	8.69	-32.33	-21.79	-30.38	-146.56	138.15	34.33	-29.34	-169.59
11	B9EIL6	-22.45	-37.3	32.59	-16.64	-20.09	-11.22	-121.27	121.1	18.5	-82.65	-176.08
12	P21453	-28.36	-0.72	24.02	15.51	-15.24	1.91	-171.08	71.06	-12.13	-86.78	-170.76
13	P48546	-13.99	-36.47	34.04	10.1	-32.32	2.81	166.03	108.59	-30.86	-61.51	-161.92
14	Q9Y5X5	-12.94	-47.29	-0.98	-50.7	-52.28	-3.96	-162.64	142.02	45.41	-12.5	-155.32
15	P29371	-10.1	-43.92	17.7	-52.36	-32.14	-5.21	-151.26	162.61	31.28	-20.09	-151.42
16	P50406	-10.14	-26.69	14.24	-10.68	-27.25	6.11	-127.21	138.72	32.25	-44	153.69
17	O95838	-13.76	-26.96	19.82	9.38	-22.69	1.17	159.01	91.62	-16.67	-57.04	-140.54
18	P35348	-12	-19.2	17.57	-2.81	-10.35	-9.9	-126.46	136.14	24.41	-69.4	179.81
19	Q6ZMH4	-24.5	-22.2	20.11	31.45	-36.06	-15.77	-137.11	145.6	7.74	-113.39	-162.88
20	P28223	-16.7	-25.99	26.52	11.37	-18.27	-27.74	-127.57	122.3	-11.04	-84.38	153.48

Table S8: **J-scores of overall, Ω type and λ type dihedral angles for computational GPCR models obtained from HGmod database**

							J-score						
**#**	**HGmod ID**	***Total***	Ω_1	Ω_2	Ω_3	Ω_4	Ω_5	Ω_6	λ_1	λ_2	λ_3	λ_4	λ_5
1	P08100	1.4	1.1	0.8	3.0	0.6	2.0	1.1	1.9	1.1	0.8	0.2	0.3
2	Q0PJU0	1.4	0.8	1.9	2.9	0.6	2.0	1.2	1.1	0.0	0.8	0.2	0.3
3	Q14332	1.7	1.7	3.4	2.3	1.7	0.6	1.2	2.2	1.2	0.1	0.2	0.3
4	P48146	1.6	1.4	2.8	2.2	1.2	0.8	0.8	3.0	0.2	0.8	0.6	0.4
5	Q6IEZ2	1.7	0.8	0.9	2.9	2.2	0.6	1.1	2.4	1.7	1.8	2.3	0.8
6	P21731	1.0	0.2	0.5	0.1	2.1	0.7	1.3	0.5	1.0	1.3	1.0	0.6
7	P58182	1.8	3.4	1.8	2.2	0.9	1.6	2.2	1.2	2.0	1.2	0.1	0.8
8	P59536	1.1	0.3	2.0	0.0	1.3	0.0	0.6	2.8	0.4	0.1	0.1	0.1
9	P0C628	2.2	6.0	1.5	0.3	0.4	1.3	1.1	2.4	0.5	1.2	1.8	1.3
10	Q5CZ62	2.0	1.3	3.5	1.9	1.8	0.8	3.7	2.3	0.6	1.0	0.4	1.1
11	B9EIL6	2.2	2.0	5.2	3.6	0.5	0.4	0.9	1.0	0.4	0.3	2.7	1.4
12	P21453	2.4	3.4	3.6	1.6	2.0	0.5	1.0	3.5	3.0	1.1	2.9	1.2
13	P48546	2.7	0.1	5.0	4.0	1.5	2.8	1.1	4.7	1.0	1.9	1.4	0.7
14	Q9Y5X5	3.6	0.4	7.6	4.2	3.2	6.5	0.1	3.1	0.8	1.5	1.4	0.4
15	P29371	2.6	1.0	6.8	0.2	3.3	2.7	0.1	2.5	1.8	0.8	1.0	0.2
16	P50406	1.5	1.0	2.6	0.6	0.1	1.8	1.6	1.3	0.6	0.9	0.4	2.9
17	O95838	2.0	0.2	2.7	0.7	1.5	0.9	0.9	5.0	1.9	1.3	1.2	0.3
18	P35348	1.1	0.6	0.8	0.1	0.5	1.4	0.7	1.3	0.4	0.5	1.9	1.6
19	Q6ZMH4	2.3	2.5	1.5	0.7	3.2	3.5	1.6	1.8	0.9	0.2	4.5	0.8
20	P28223	2.0	0.6	2.4	2.2	1.6	0.1	3.3	1.3	0.3	1.1	2.8	2.9

Table S9: PDBs and dihedral angles of active-like GPCR Class A structures from PDB.

#	PDB	Ω_1	Ω_2	Ω_3	Ω_4	Ω_5	Ω_6	λ_1	λ_2	λ_3	λ_4	λ_5	Subfamily
1	4UHR	-15.9	-24.1	20.1	-0.1	-21.7	-8.5	-120.6	144.5	2.4	-63.9	162.3	Adenosine receptor A2
2	3SN6R	-15.1	-15.9	18.88	-20.1	-20.2	-18.4	-103.1	140.7	28.4	-60.8	177.4	β2 adrenergic receptor
3	5GLHA	-13.1	-21.8	12.87	-18.1	-26.1	-4.3	-81.7	129.9	33.1	-44.8	176.1	Endothelin
4	4MQSA	-21.8	-16.2	18.49	3.2	-14.0	-15.1	-109.4	123.6	-5.2	-80.9	169.2	Muscarinic (Acetylcholine)
5	4GRVA	-10.1	-16.3	14.28	-33.5	-18.4	-17.7	-101.2	137.5	39.2	-39.5	133.1	Neurotensin
6	4PXF	-11.5	-14.7	24.61	-9.9	-21.3	-6.7	-109.3	132.8	18.7	-66.5	171.9	Purinoreceptor
7	4X1H	-14.5	-16.3	20.37	-11.9	-21.9	-6.5	-141.5	126.5	36.1	-67.3	163.5	Rhodopsin
8	4XT1A	-12.7	-14.2	15.76	-20.6	-19.4	-12.6	-155.5	95.5	21.4	-56.1	168.4	Viral GPCR US28
9	5C1MA	-21.4	-22.1	14.13	-14.0	-16.7	-17.3	-120.4	125.9	25.6	-51.7	173.1	Opioid mu receptor
10	4IB4A	-12.2	-17.1	10.62	-6.9	-10.8	-5.6	-107.1	126.2	-7.1	-42.7	151	5-hydroxytryptamine receptor 2B

Figure S1: Arrangements of the helices and loops depending on the Ω and λ dihedral angles. The central figure presents the front view of three consecutive helices in the membrane proteins. $H_{(n)}$ represents the helices and $L_{(n)}$ represents loops. (A) Front view of the arrangement of two adjacent helices, H_i and H_{i+1} when $\Omega_i = 0^\circ$, (B) Front view of the arrangement of two adjacent helices, H_i and H_{i+1} when $\Omega_i = \pm 180^\circ$, (C) Top view of the arrangement of two adjacent loops, L_i and L_{i+1}, and three adjacent helices, H_i, H_{i+1} and H_{i+2}, when $\lambda_i = 0^\circ$, and (D) Top view of the arrangement of two adjacent loops, L_i and L_{i+1}, and three adjacent helices, H_i, H_{i+1} and H_{i+2}, when $\lambda_i = \pm 180^\circ$. These figures show the possible scenario depending on the dihedral angles rather than a real arrangement observed in TM proteins.

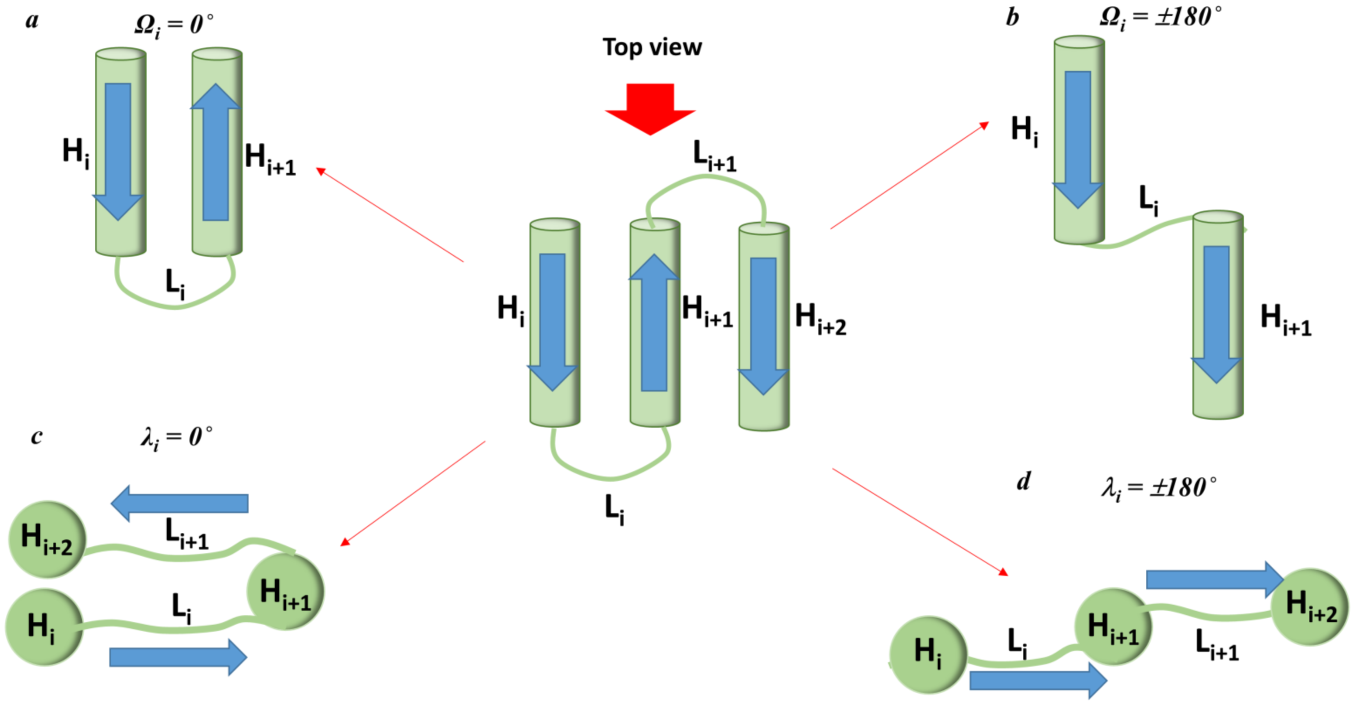

Supplement: Supplementary file 1 — Supplementary Information [file 41598_2017_15513_MOESM1_ESM.pdf]
